# Direct observation of Cu in high-silica chabazite zeolite by electron ptychography using Wigner distribution deconvolution

**DOI:** 10.1038/s41598-023-27452-3

**Published:** 2023-01-06

**Authors:** Kazutaka Mitsuishi, Katsuaki Nakazawa, Ryusuke Sagawa, Masahiko Shimizu, Hajime Matsumoto, Hisashi Shima, Takahiko Takewaki

**Affiliations:** 1 Research Center for Advanced Measurement and Characterization, National Institute for Materials Science, 1-2-1 Sengen, Tsukuba, Ibaraki 305-0047 Japan; 2 International Center for Young Scientists (ICYS), National Institute for Materials Science, 1-2-1 Sengen, Tsukuba, Ibaraki 305-0047 Japan; 3 JEOL Ltd., 3-1-2 Musashino, Akishima, Tokyo 196-8558 Japan; 4 Materials Characterization Laboratory, Mitsubishi Chemical Corporation, 1000 Kamoshida-cho, Aoba-ku, Yokohama-shi, Kanagawa Japan; 5 Science and Innovation Center, Mitsubishi Chemical Corporation, 1000 Kamoshida-cho, Aoba-ku, Yokohama-shi, Kanagawa Japan

**Keywords:** Catalysis, Microscopy

## Abstract

Direct observation of Cu in Cu-chabazite (CHA) zeolite has been achieved by electron ptychography using the Wigner distribution deconvolution. The imaging properties of ptychographically reconstructed images were evaluated by comparing the intensities of six-membered-ring columns of the zeolite with and without Cu using simulated ptychography images. It was concluded that although false contrast may appear at Cu-free columns for some acquisition conditions, ptychography can discriminate columns with and without Cu. Experimental observation of CHA with and without Cu was performed. Images obtained from the Cu-containing sample showed contrast at the six-membered-rings, while no contrast was observed for the Cu-free sample. The results show that ptychography is a promising technique for visualizing the atomic structures of beam-sensitive materials.

## Introduction

As environmental awareness increases, automobile exhaust gas emissions and fuel-consumption regulations are becoming increasingly stringent. Zeolites are mainly used as urea-selective catalytic reduction (SCR) catalysts for nitrogen oxide (NO_*x*_) purification in diesel engines^1,2^. SCR systems using iron-supported zeolite catalysts were first commercialized. Subsequently, copper-supported zeolite catalysts with higher performance and durability have been developed.

Copper-supported zeolite catalysts are preferred to unsupported eight-membered-ring zeolites, such as chabazite (CHA)-type zeolites^3,4^. Cu-CHA catalysts effectively purify NO_*x*_ even at temperatures as low as 200 °C and can withstand high-temperature steam at 700–900 °C^5,6^. Research is underway to improve the performance and durability of copper-supported zeolite catalysts and to reduce N_2_O emissions, which have a very high global warming potential. Hence, it is necessary to elucidate the mechanism of the SCR reaction, which has been studied using various spectroscopic and computational methods^6,7^. It is necessary to determine the positions of the Cu ions, the catalytically active species, in zeolites. Furthermore, observation of the Cu-ion state in the reaction field will clarify the mechanism of activity onset and degradation, leading to the development of more efficient SCR catalysts, and also to the application of copper-loaded AEI-type zeolites, which are another promising SCR catalyst^7^. In addition to SCR reactions, determination of the positions of the Cu ions in zeolites is expected to facilitate other important reactions, such as the direct NO_*x*_ decomposition and methanol synthesis by methane oxidation. However, even in its non-activated state, direct observation of the Cu^2+^ positions in small-pore CHA has not been achieved. This is because zeolites are very beam-sensitive materials. Consequently, it is difficult for direct imaging methods to identify the location of Cu, which typically has a very small site occupancy of approximately 0.05^8^.

Transmission electron microscopy (TEM) has long been a method of choice for direct imaging of atoms. In particular, annular dark field scanning TEM (ADF-STEM)^9–11^ has been widely used for the last 20 years. In ADF-STEM, thermal diffuse scattering (TDS) electrons are summed with an annular detector. The resultant image is considered to be incoherent, and no contrast reversal occurs, that is, atom always appear as bright contrast, and hence images are easy to interpret. On the other hand, the use of TDS electrons makes ADF-STEM very dose inefficient, because most of the electrons are weakly scattered and only a very small portion of the electrons undergo TDS.

With the recent developments of detector technology, new imaging modes of TEM are emerging. For example, four-dimensional STEM (4D-STEM)^12^ uses a fast pixelated detector to acquire the diffraction pattern during STEM acquisition, and a variety of reconstruction methods, such as differential phase contrast (DPC) imaging^13–16^, optimized bright-field imaging^17,18^, integrated DPC imaging^19–22^ and ptychography^23–26^, can be used to reconstruct the image. In contrast to ADF-STEM that preferentially selects electrons that scatter to a large angle by TDS, 4D-STEM can employ the interference between weakly scattered electrons, and thereby greatly increase the dose efficiency. For example, integrated DPC-STEM has been successfully used to visualize Fe and Mo atoms in zeolite structures^20,27^.

Among the many 4D-STEM techniques, ptychography is particularly promising. Since its original proposal by Hoppe in 1969^28^, it has been progressed very much and many interesting results are emerging. Ptychographic reconstruction of STEM data can be performed using a “direct method” or an “iterative method”^29^. The “direct method”, which is also called “focused probe ptychography”, uses a focused probe with very dense sampling in real space, and the reconstruction is performed with the Wigner distribution deconvolution (WDD)^30^ or the single side band (SSB) method^24^. Although the data size is huge, the solution can be obtained in a quick and deterministic way with a set of linear transformations^31^. In the “iterative method”, the phases are reconstructed by iterative algorithms such as ptychographical iterative engine (PIE)^32,33^. In PIE, the phase is derived in the same manner as in diffractive imaging^34,35^, using overlapping regions between positions as constraints during the iterative cycles, rather than the support areas around the sample. By assuming multiple object slices upon reconstruction, it is possible to reconstruct three-dimensional objects^36^.

In ptychography, in addition to its high dose efficiency, aberrations (including defocus) can be calculated and removed after acquisition^37^. Therefore, fine adjustments are not necessary before the acquisition, and the irradiation dose can be significantly reduced. However, ptychography uses coherent interference, and its imaging properties are not yet fully understood.

In this study, we report direct observation of Cu atoms in Cu-high-silica-CHA (SSZ-13) zeolite, by focused probe electron ptychography combined with WDD reconstruction and multi-slice-based simulations^38^. For a first trial, the observation was performed at room temperature without any activation gas. Under this condition, it is expected that Cu is located at the six-membered rings on the top and bottom of the eight-membered-ring pore cage^39,40^. The contrast of the six-membered-ring columns obtained by electron ptychography was compared with simulations, revealing that ptychography can visualize Cu in Cu-SSZ-13 under a wide range of acquisition conditions.

## Results

### Visualization of Cu atom at the six-membered-ring pore in CHA

**Figure 1:**
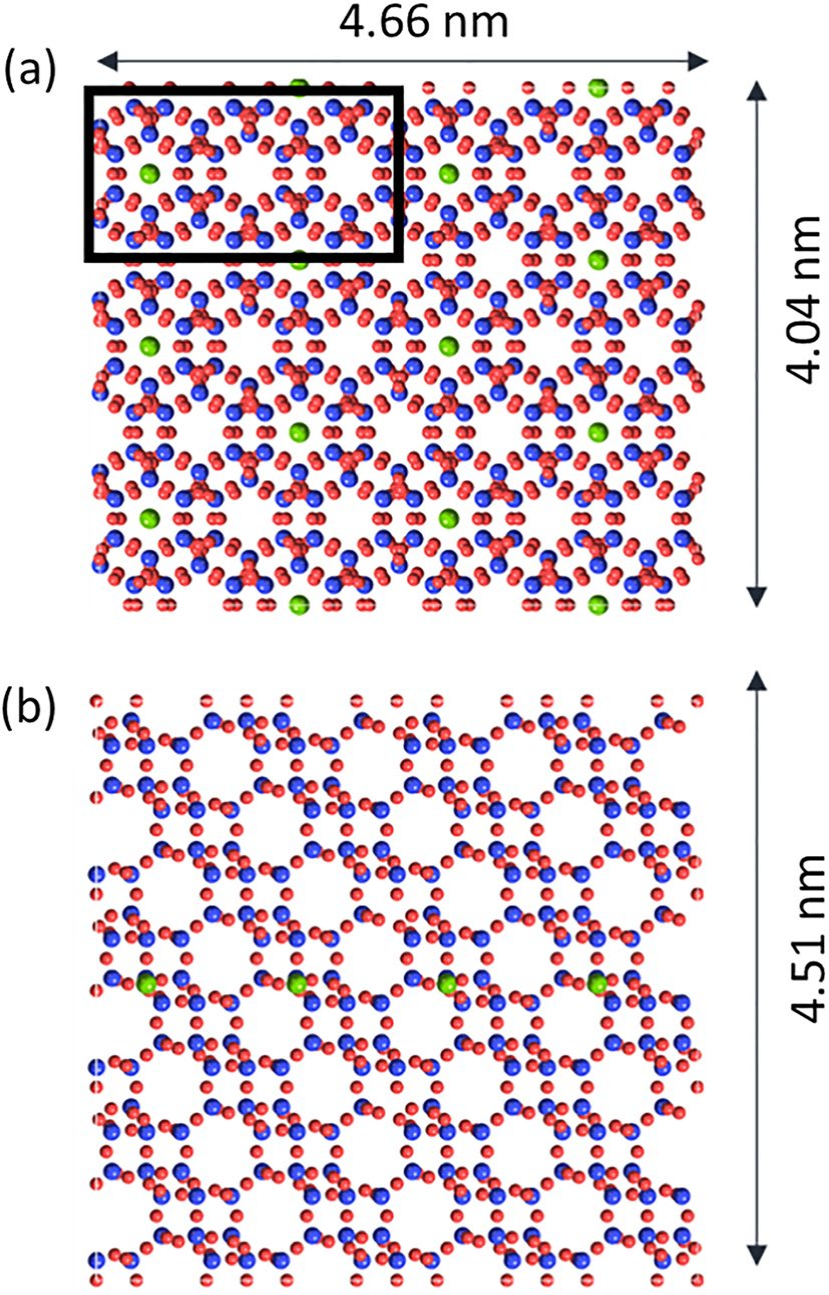
Supercell used for ptychographic image simulation viewed from the (**a**) *x–y* and (**b**) *y*–*z* directions. Cu, Si, and O atoms are green, blue, and red, respectively. The black rectangle in (**a**) contains a six-membered-ring column with Cu (left) and two six-membered-ring columns without Cu (center and right).

In contrast to ADF-STEM, ptychography uses coherent interference to form its image, and it may be susceptible to contrast changes when varying the imaging conditions. Moreover, ptychography can acquire data with an aberrated probe (e.g., at non-zero defocus) and then estimate and remove the aberrations after acquisition. The capability of aberration correction depends on the resolution of recorded diffraction patterns, since the phase error due to probe aberration cause inhomogeneity of the phase at the transmitted disk, and the largest phase change that can be properly recorded by a pixelated detector is limited to less than 2π between the neighboring pixels. However, a more important concern is the coherent imaging nature of ptychography. Hence, as in high-resolution TEM, image artifacts may occur in ptychography at relatively small defocus values, and it is not clear whether aberration-corrected images are the same with the one obtained without aberrations. To clarify this point, and to discuss visualization of Cu, through-focus image simulations were performed for different defocus pairs of acquisition and reconstruction. In this way we simulated the situation in which the data were acquired with a defocused probe, and then the defocus was compensated for upon reconstruction. To observe the contrast difference between the columns with and without Cu, six-membered-ring columns with and without Cu were constructed as simulation models (Fig. 1). To calculate the expected Cu contrast relatively to the contrast of the other atoms, the thickness of the supercell was set so that each six-membered-ring viewed from the [0001] direction contained one Cu atom on average, which was expected from the Cu concentration in our samples. For the columns with Cu atoms, the Cu atoms were placed at the center of the six-membered rings and at the middle of the simulated slab, which had a thickness of 4.5 nm. The effect of the convergence semi-angle of the probe was studied using two values of 13.5 and 20.8 mrad.

**Figure 2:**
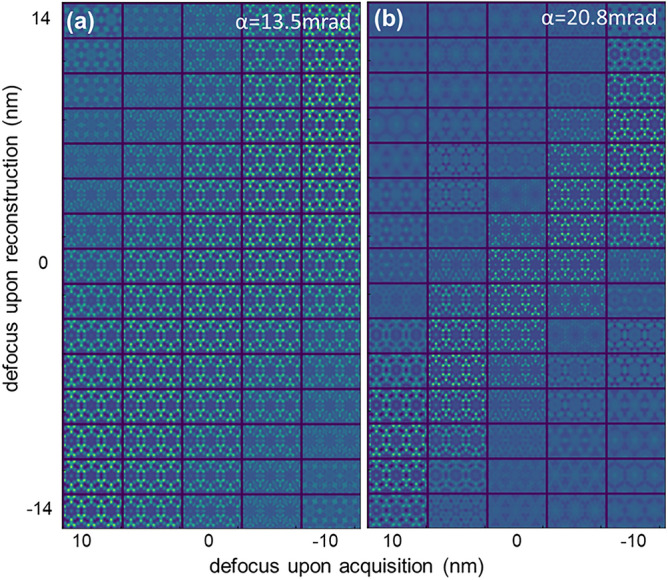
Simulated images of the black rectangle region in Fig. 1a containing six-membered-ring columns with (left) and without (center and right) Cu for the pairs of acquisition (horizontal) and reconstruction (vertical) defocus values with convergence semi-angles of (**a**) 13.5 mrad and (**b**) 20.8 mrad. The simulated area is 2.3 nm × 1.3 nm.

Figure 2a,b show the through-focus images of the black rectangle region in Fig. 1, which contained a six-membered-ring column with Cu (left column) and two six-membered-ring columns without Cu (center and right columns); the images were calculated for different defocus values and convergence semi-angles of 13.5 and 20.8 mrad, respectively, and reconstructed by the WDD method^30,41^. The horizontal axis shows defocus during acquisition (+ 10 nm to − 10 nm), and the vertical axis presents defocus that was set upon reconstruction (− 14 nm to 14 nm).

It is interesting to note that the image contrast was high in a diagonal strip running from the top right to the bottom left of the defocus pairs of acquisition and reconstruction. For example, for the data acquired at 0 nm defocus, the contrast was high around 0 nm defocus for reconstruction, and for the data acquired at 10 nm (over focus), the highest contrast appeared around − 10 nm for reconstruction. This contrast behavior can be qualitatively understood as follows: the defocus causes a parabolic phase difference relatively to the distance from the center of the transmitted disk captured by the pixelated detector. Therefore, when the data were acquired at zero defocus, the phases of different pixel positions became in phase with zero defocus in reconstruction, resulting in a high-contrast phase image. When the defocus value was non-zero upon reconstruction, the phases of the different pixel positions became out of phase, thereby reducing the image contrast. For the data acquired at finite defocus values, the contrast will be maximized when an appropriate phase is added by applying some defocus upon reconstruction that compensates for the defocus upon acquisition. It can be clearly seen that in the high-contrast region of the defocus pair, the zeolite cage structures were reconstructed well even from the data acquired with defocused probe, proving the aberration-correction capability of ptychography, and indicating that the contrast can be used as a simple indicator of the appropriate defocus.

Careful inspection of the figure reveals that the strip of highest contrast is slightly shifted downward, such that for zero acquired defocus, the highest contrast appeared at approximately − 2 nm reconstruction defocus. This is because of the thickness of the supercell (4.5 nm); hence the effective in-focus position was approximately the − 2 nm defocused position, similar to the case of DPC which shows the highest contrast when the probe is focused near the middle of the sample^18^.

The difference between the two convergence semi-angles was not obvious, because 13.5 mrad was already sufficiently large to resolve the cage structure of zeolite. However, the width of the high-contrast strip region of the defocus pair was wider for a smaller convergence angle, because the depth of focus is shorter for a larger convergence semi-angle.

**Figure 3:**
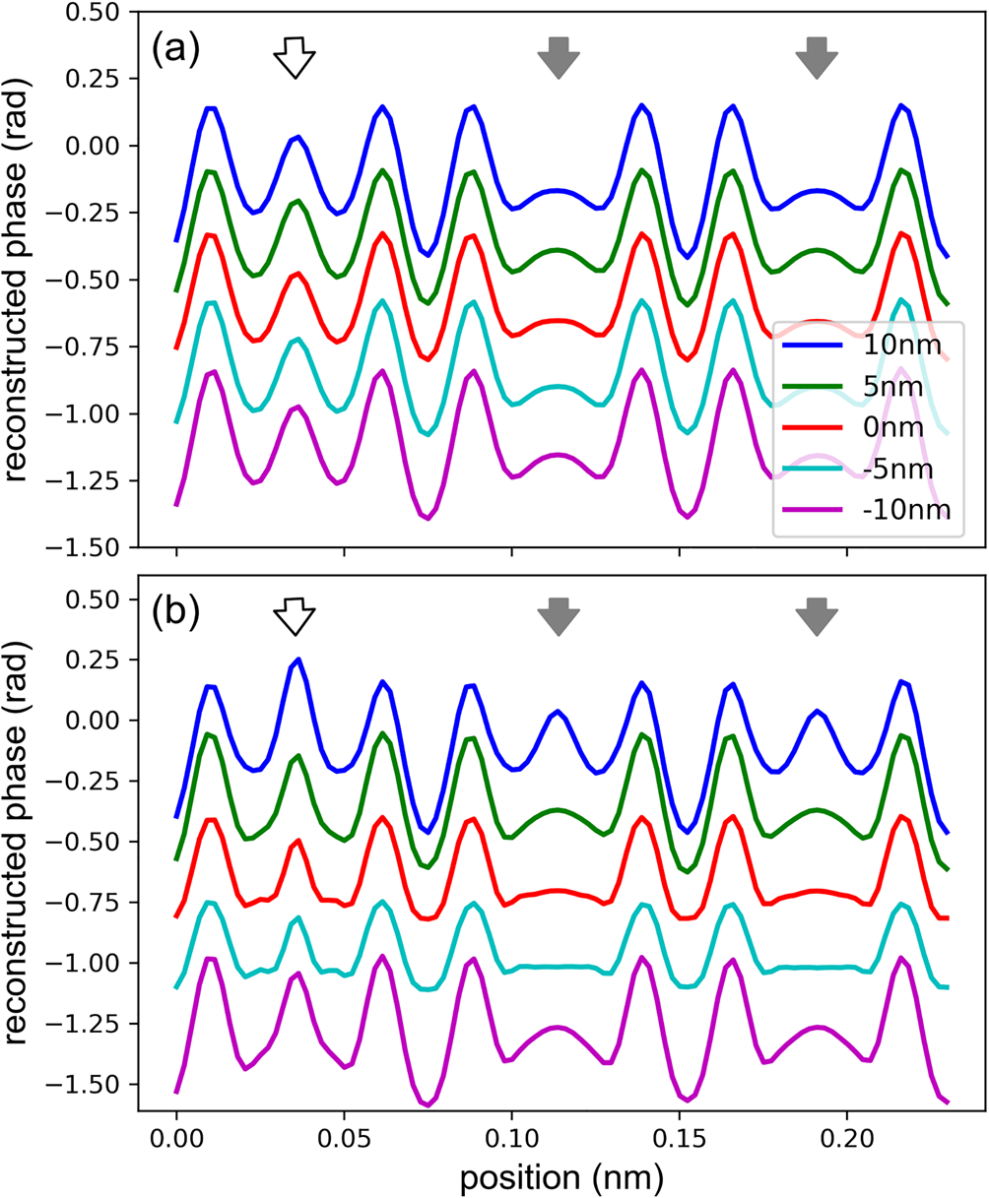
Profiles taken along the line at the center of black rectangle in Fig. 1a for the acquisition defocuses of 10, 5, 0, − 5, and − 10 nm and the convergence semi-angles of **a** 13.5 mrad and (**b**) 20.8 mrad. The white and gray arrows mark the centers of six-membered ring columns with and without Cu, respectively. The reconstruction defocus is chosen so that it compensates for the acquisition defocus. The profiles are vertically shifted for display purpose.

To clarify the Cu contrast we plotted in Fig. 3a line profiles taken at the center of black rectangle in Fig. 1a for the acquisition defocus values of 10, 5, 0, − 5, and − 10 nm. The white and gray arrows indicate the positions of the center of six-membered ring columns with and without Cu, respectively. The reconstruction defocus is chosen so that it compensates for the acquisition defocus. The profiles are vertically shifted for clarity of presentation.

The line profiles confirm that the phase value at the center of six-membered ring is higher for a ring with Cu (white arrow) than without Cu (gray arrows) for all acquisition defocus values, and the difference in contrast is sufficiently high to identify Cu atoms. This result shows that, if properly corrected, the acquisition defocus does not affect the result within this acquisition defocus range (± 10 nm) and we can safely attribute the bright contrast inside the six-membered rings to Cu. The intensity minima between the oxygen columns are lower than the minima in the six-membered ring columns without Cu, which is counterintuitive because the atomic potential should be smaller at the ring column positions. This effect is more prominent for a large defocus, and thus it may, at least partly, originate from dynamical scattering. This is because WDD reconstruction does not consider multiple scattering, and because psychography is a coherent imaging technique which is susceptible to contrast changes, like high-resolution TEM. Hence image simulation is essential for ptychographical analysis.

The profiles for other defocus combinations are presented in the Supplementary Fig. S2 for a convergence semi-angle of 13.5 mrad. For a zero acquisition defocus, the intensity changes across a six-membered ring without Cu were small for a wide range of reconstruction defocus values, as confirmed from the third column of Fig. S2a,d. Hence the columns with and without Cu can be clearly distinguished. However, false contrast appears in Cu-free columns even at a relatively small acquisition defocus of 10 nm (Fig. S2b). Although it is not obvious, this can also be seen in the left column of Fig. 2a, especially for a larger defocus value than the value used to compensate for the defocus upon acquisition. This means that although the cage structure of zeolite can be reproduced well from the data acquired under the defocused condition, there is a potential risk of false contrast for low-occupancy sites. However, for a small convergence semi-angle of 13.5 mrad, the false contrast was weak, and could be distinguished from a genuine Cu contrast.

The situation was more problematic for a large convergence semi-angle of 20.8 mrad. Figure 3b shows the line profiles taken at the center of black rectangle in Fig. 1a for the acquisition defocus values of 10, 5, 0, -5, and -10 nm and a convergence semi-angle of 20.8 mrad. The white and gray arrows indicate the positions of the center of six-membered ring columns with and without Cu, respectively. The reconstruction defocus is chosen so that it compensates for the acquisition defocus. The profiles are vertically shifted for clarity of presentation.

The line profiles across the six-membered-rings without Cu are flatter, and the Cu profile is sharper for the data acquired at zero defocus and 20.8 mrad as compared to 13.5 mrad. However, the false contrast at 10 nm defocus is more prominent for 20.8 mrad (cf. blue lines in Fig. 3a,b). This effect can also be seen in the first column of Fig. 2b. It can be explained by a shorter depth of focus for a larger convergence semi-angle; hence a small deviation of the reconstruction defocus causes a stronger false contrast. The profiles for other defocus combinations are presented in the Supplementary Fig. S3 for a convergence semi-angle of 20.8 mrad.

### Dose dependence of the Cu visibility

Zeolites are easily damaged by electron beam, and hence it is important to estimate the minimum electron dose that would still allow detection of Cu in six-membered-ring columns. In Supplementary Figure S4 we present simulated images for reconstruction defocus ranging from − 14 to 14 nm and irradiation dose varied from 5 to 5000 electrons per scan point. The effect of the dose was simulated by a Poisson distribution, and the convergence semi-angle was set to 13.5 mrad. The relation between e^−^/point and e^−^/Å^2^ is as follows: a dose of 100 e^−^/point with 512 × 512 scan points for 10 nm × 10 nm scan area results in 2600 e^−^/Å^2^.

It is surprising that the six-membered-ring structure can be discerned even with 5 e^−^/point, which means only five electrons in each diffraction image captured by the pixelated detector, corresponding to 130 e^−^/Å^2^. However, reliable detection of the Cu site requires a dose of at least 50 e^−^/point.

### Experimental observation of Cu in Cu-SSZ-13

**Figure 4:**
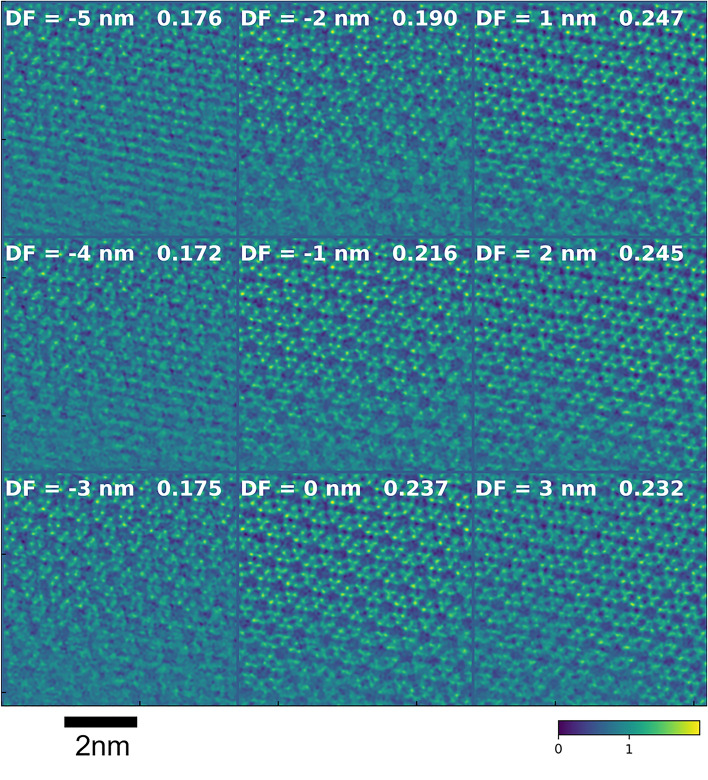
Experimental images of Cu-containing CHA reconstructed with defocus ranging from − 5 to 3 nm. The number at the top right of each image is the standard deviation of the image.

**Figure 5:**
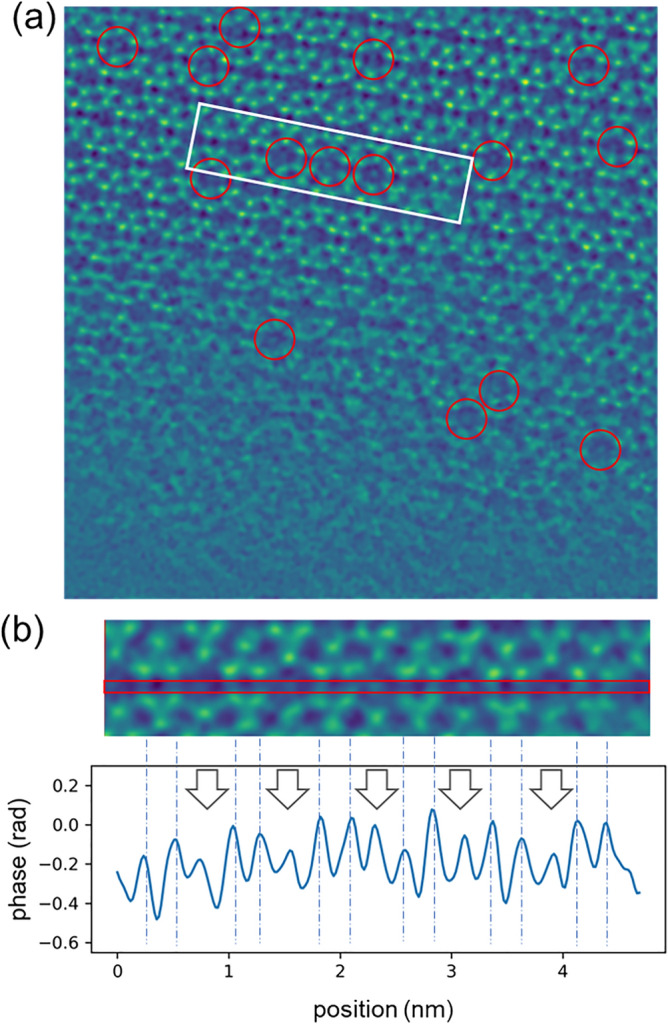
An image of Cu-containing CHA reconstructed with zero defocus. Red circles mark selected six-membered rings that contain Cu atoms with a high probability. (**b**) Line profile taken from the center of the white rectangle in (**a**). The arrows in (**b**) indicate the centers of six-membered rings, and the dotted lines mark the oxygen columns.

Based on the above simulation results, a convergence semi-angle of 13.5 mrad and a probe current of 0.096 pA were chosen to acquire experimental data from CHA with and without Cu. The through-focus images of Cu-CHA reconstructed with a defocus of − 5 nm to + 3 nm are shown in Fig. 4. To compare the image contrast, the standard deviation of each image is also shown in Fig. 4. The current of 0.096 pA corresponds to approximately 80 e^−^/point and 2100 e^−^/Å^2^. At this current it was not possible to accurately adjust the focus and astigmatism before the image acquisition and to see atomic details during the acquisition. The focus and astigmatism were estimated by the method suggested by Yang et al.^37^ and removed together with other aberrations upon reconstruction. The measured aberrations are listed in Supplementary Table S1. The estimated defocus was − 1.68 nm, which should be small enough to qualify for in-focus condition for the convergence semi-angle of 13.5 mrad. Nevertheless, we have compensated for this small defocus before the through-focus calculation. Figure 5a shows the reconstructed image, where we have marked the Cu atoms by red circles. The noise and distortions in the image originate from detector electronics, charging effects, and sample drift during acquisition, which took approximately 35 s for 512 × 512 scan points. Despite the noise, the cage structure of the zeolite was clearly visualized and the contrast at the center of six-membered ring was obvious. Figure 5b shows a line profile taken between arrows in Fig. 5a. The profile was averaged over a width of 5 pixels. The arrows in Fig. 5b indicate the centers of six-membered rings, and the dotted lines mark the oxygen columns. Clear peaks are observed at the center of six-membered rings, in a close agreement with the simulations. As shown in Fig. 5, the Cu contrast was observed for a wide range of reconstruction defocus values. Note that the Cu atoms did not always appear at the center of six-membered rings, but often shifted in various directions, and systematic investigation of this effect would be an interesting topic for a separate study.

**Figure 6:**
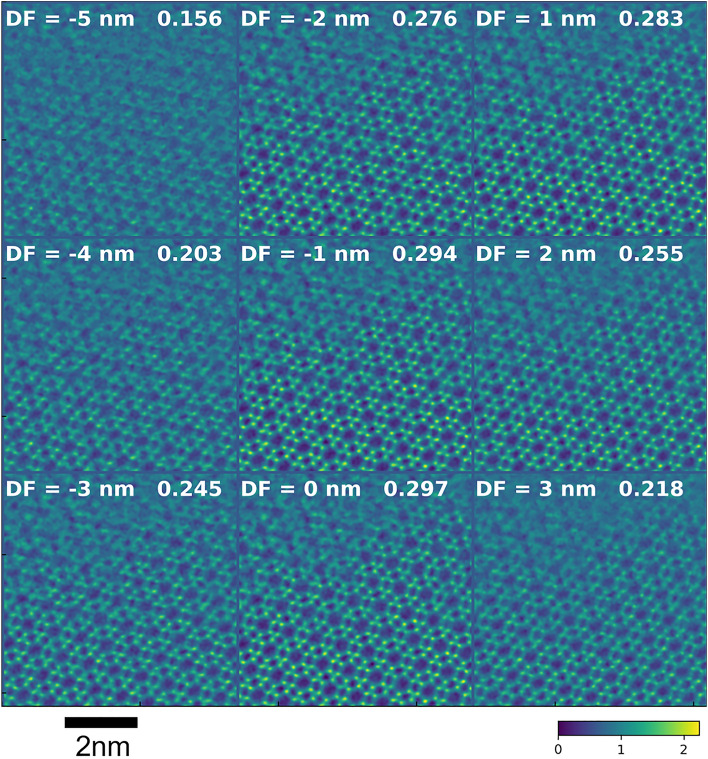
Experimental images of Cu-free CHA reconstructed with defocus ranging from − 5 to 3 nm. The number at the top right of each image is the standard deviation of the image.

**Figure 7:**
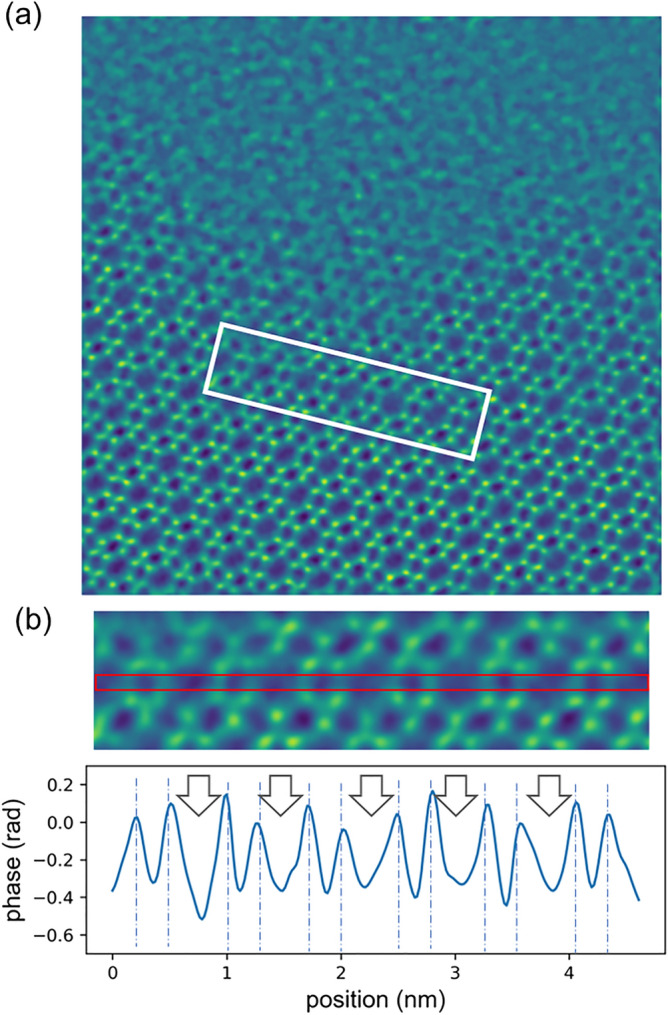
An image of Cu-free CHA reconstructed with zero defocus. (**b**) Line profile taken from the center of the white rectangle in (**a**). The arrows in (**b**) indicate the centers of six-membered rings, and the dotted lines mark the oxygen columns.

Figures 6 and 7a show through-focus images of Cu-free CHA for a 13.5 mrad convergence semi-angle, which were reconstructed with defocus values of − 5 nm to + 3 nm and zero, respectively. The experimental defocus was estimated at 1.50 nm (See Supplementary Table S1) and compensated for during the reconstruction. The zeolite structure is clearly visible, as in Figs. 4 and 5, but the bright contrast at the center of the six-membered rings was not observed for a wide range of reconstruction defocus values. Figure 7b shows a line profile taken between arrows in Fig. 7a. The profile was averaged over a width of 5 pixels. The arrows in Fig. 7b indicate the centers of six-membered rings, and the dotted lines mark the oxygen columns. Contrary to Fig. 5b no peaks are observed at the centers of six-membered rings. From those results, we conclude that ptychography did visualize dispersed Cu atoms, a catalytically active species in a beam-sensitive zeolite material.

## Conclusions

Detectability of Cu atoms in Cu-CHA by electron ptychography has been discussed by comparing the intensities of columns with and without Cu using multi-slice-based simulations. The simulations were carried out for two convergence semi-angles (13.5 and 20.8 mrad) and different defocus conditions. The zeolite cage structure could be well visualized from the data acquired under defocus conditions; however, false contrast appeared at columns without Cu. The false intensity was more prominent for the larger convergence semi-angle (20.8 mrad). Experimental images of SSZ-13 zeolite with and without Cu were recorded with a smaller convergence semi-angle (13.5 mrad) and the data were obtained almost in focus. Ptychography can evaluate the aberrations after acquisition, and thus we have checked the defocus values. The obtained images showed atomic contrast at the six-membered-ring positions for the sample with Cu, while no contrast was observed from the Cu-free sample. We have demonstrated that because of its high beam efficiency, ptychography can directly visualize Cu atoms in beam-sensitive materials such as zeolites. By extending this method to in situ measurement of activated states, it will be possible to directly visualize the SCR process.

## Methods

### SSZ-13 zeolite synthesis

Solution A was prepared by adding aluminum hydroxide to an aqueous solution of NaOH and KOH and stirring until the dissolution of aluminum hydroxide. *N*,*N*,*N*-trimethyl-1-adamantammonium hydroxide (TMAdaOH, 25 wt%) and CATALOID SI-30 (SiO_2_, 30 wt%) were added to solution A, and the solution was stirred for 1 h, followed by addition of seeds (SSZ13, 2 wt% with respect to the SiO_2_ source) and stirring for another hour. The mixture with a chemical composition of 0.033 Al_2_O_3_/SiO_2_/0.1 NaOH/0.06 KOH/0.07 TMAdaOH/20H_2_O/seeds (2%) (total 101 kg) was hydrothermally treated in a 100-L stainless-steel vessel at 150 °C for 48 h while stirring at 100 rpm. The solid product was separated and washed thoroughly with deionized water. The obtained solid was dried, giving the as-synthesized zeolite sample. To remove the organic structure-directing agent from the sample, the as-synthesized zeolite was calcined in air at 600 °C for 6 h, using a heating rate of 1 °C min^−1^, giving the calcined zeolite sample. As-calcinated zeolites contains a mixture of Na^+^, K^+^ and H^+^ ions. The proton ion-exchanged sample was chosen as a Cu-free control sample.

### Proton ion-exchange

The calcined zeolite (12 g) was dispersed in NH_4_NO_3_ aqueous solution (240 mL, 1.2 mol L^−1^) at 80 °C for 2 h. This treatment was performed twice. After ion exchange, the sample was well-rinsed with distilled water at room temperature and dried overnight at 100 °C under air using a conventional oven. The obtained NH_4_ form of the zeolite was calcined at 550 °C for 3 h, giving the H-form zeolite.

### Copper ion exchange

The H-form zeolite (10.5 g) was dispersed in Cu(CH_3_COO)_2_ aqueous solution (105 mL, 0.05 mol L^−1^) at 50 °C for 2 h. This treatment was performed twice. After ion exchange, the sample was well-rinsed with distilled water at room temperature and dried overnight at 100 °C under air using a conventional oven. The obtained Cu form of the zeolite was calcined at 500 °C for 2 h, giving the Cu-form zeolite. The SiO_2_/Al_2_O_3_ and Cu/Al ratios of the obtained Cu-form zeolite were determined to be 25.2 and 0.56 by X-ray fluorescence analysis, respectively. The Cu/Al value of 0.57 was higher than the theoretical upper limit of 0.5 assuming Cu^2+^, so presumably some Cu segregated on the surface as Cu oxides.

Spatial distribution of Cu in samples with and without Cu was measured by energy-dispersive X-ray spectroscopy (EDS) in a scanning electron microscope (SEM). The obtained spatial maps and spectra (Supplementary figures S5 and S6) reveal a homogeneous distribution of Cu for Cu-exchanged sample, while no Cu signal was observed for the proton-exchanged material.

### Ptychography observations

The samples with and without Cu were crushed in an agate mortar with ethanol and dispersed on TEM microchips of the in situ heating holder (Protochips Fusion Select; see also Supplementary Figure S1). Before observation, the microchips were heated at 400 °C in vacuum for 30 min to remove water and other contaminants. The ptychography observations were performed at room temperature and 200 kV with an aberration-corrected transmission electron microscope (JEOL JEM-ARM200F) equipped with a fast pixelated STEM detector (JEOL 4DCanvas™). For the convergence semi-angle of 13.5 mrad, the corresponding probe current was 0.096 pA. The diffraction patterns were acquired with 66 × 66 pixels, and the camera length was set so that the transmitted disk filled approximately 80% of the detector, which corresponds to 0.5 mrad/pixel. The frame rate was fixed at 7500 frames per second.

Ptychographic reconstruction was performed by the WDD method^30^ using a custom-built Python code. Post-aberration estimation and correction were performed by the method proposed by Yang et. al.^37^.

### Ptychographic image simulations

For the ptychographic image simulations, the diffraction pattern of each probe position of 4D-STEM was calculated by multi-slice simulations using the abTEM code developed by Madsen and Susi^42^. For the multi-slice simulations, the SSZ-13 unit-cell structure was extended in the *x*, *y*, and *z* directions and cut to make a rectangular supercell of 4.0 nm × 4.7 nm × 4.5 nm (Fig. 1). To calculate the expected Cu contrast relatively to the contrast of other atoms, the thickness of the supercell was determined such that each six-membered-ring viewed from the [0001] direction contained one Cu atom on average, which is expected from the Cu concentration in our samples. That is, for the SiO_2_/Al_2_O_3_ ratio of 25.2, and assuming that the Cu/Al ratio is 0.5, a Cu atom exists in approximately 1 out of every 4.3 six-membered rings. Thus, if we take 3-unit-cell thickness, every six-membered-ring column viewed from the [0001] direction should contain one Cu atom on average. To observe the contrast difference between the columns with and without Cu, six-membered-ring columns with and without Cu were constructed in the supercell. For the columns with Cu atoms, the Cu atoms were placed at the center of the six-membered rings and at the middle of the supercell thickness. All tetrahedral sites were modeled as silicon atoms because substitution of a Si atom with an Al atom results in a very small phase difference. The irreducible region of the model supercell (the rectangular region in Fig. 1) was chosen as the scan region, and the obtained diffraction patterns were tiled to make 512 × 512 input arrays for ptychographic reconstruction. The number of scan steps and detector pixel size were the same as in experiment. Electron absorption and TDS were ignored.

## Supplementary Information

Supplementary Information.

## Data Availability

All relevant data are available from the corresponding authors upon reasonable request.

## Code availability

The code used for WDD reconstruction and aberration estimations is available at GitHub https://github.com/Kazu32143952/Ptychography-reconstruction-by-WDD-method-and-aberration-measurement.

## References

[1] Beale AM, Gao F, Lezcano-Gonzalez I, Peden CH, Szanyi J (2015). Recent advances in automotive catalysis for NOx emission control by small-pore microporous materials. Chem. Soc. Rev..

[2] Han L (2019). Selective catalytic reduction of NOx with NH3 by using novel catalysts: State of the art and future prospects. Chem. Rev..

[3] Fickel DW, D’Addio E, Lauterbach JA, Lobo RF (2011). The ammonia selective catalytic reduction activity of copper-exchanged small-pore zeolites. Appl. Catal. B.

[4] Zhang L (2019). Recent advances in the preparation of zeolites for the selective catalytic reduction of NOx in diesel engines. React. Chem. Eng..

[5] Borfecchia E (2018). Cu-CHA—A model system for applied selective redox catalysis. Chem. Soc. Rev..

[6] Lee H, Song I, Jeon SW, Kim DH (2021). Mobility of Cu ions in Cu-SSZ-13 determines the reactivity of selective catalytic reduction of NOx with NH3. J. Phys. Chem. Lett..

[7] Oda A, Shinoya H, Hotta Y, Takewaki T, Sawabe K, Satsuma A (2020). Spectroscopic evidence of efficient generation of dicopper intermediate in selective catalytic reduction of NO over Cu-ion-exchanged zeolites. ACS Catal..

[8] Fickel DW, Fedeyko JM, Lobo RF (2010). Copper Coordination in Cu-SSZ-13 and Cu-SSZ-16 Investigated by Variable-Temperature XRD. J. Phys. Chem. C.

[9] Pennycook SJ, Jesson DE (1991). HIgh-resolution Z-Contrast imaging of crystals. Ultramicroscopy.

[10] Nellist PD, Pennycook SJ (1999). Incoherent imaging using dynamically scattered coherent electrons. Ultramicroscopy.

[11] Loane RF, Xu P, Silcox J (1992). Incoherent imaging of zone axis crystals with Adf stem. Ultramicroscopy.

[12] Ophus C (2019). Four-Dimensional Scanning Transmission Electron Microscopy (4D-STEM): From scanning nanodiffraction to ptychography and beyond. Microsc. Microanal..

[13] Rose H (1977). Nonstandard imaging methods in electron microscopy. Ultramicroscopy.

[14] Dekkers NH, Lang HD (1974). Differential phase-contrast in a stem. Optik.

[15] Shibata N (2012). Differential phase-contrast microscopy at atomic resolution. Nat. Phys..

[16] Kohno Y, Seki T, Findlay SD, Ikuhara Y, Shibata N (2022). Real-space visualization of intrinsic magnetic fields of an antiferromagnet. Nature.

[17] Ooe K, Seki T, Ikuhara Y, Shibata N (2019). High contrast STEM imaging for light elements by an annular segmented detector. Ultramicroscopy.

[18] Ooe K, Seki T, Ikuhara Y, Shibata N (2021). Ultra-high contrast STEM imaging for segmented/pixelated detectors by maximizing the signal-to-noise ratio. Ultramicroscopy.

[19] Lazic I, Bosch EGT, Lazar S (2016). Phase contrast STEM for thin samples: Integrated differential phase contrast. Ultramicroscopy.

[20] Liu D (2021). Possible misidentification of heteroatom species in scanning transmission electron microscopy imaging of zeolites. J. Phys. Chem. C.

[21] Shen B, Chen X, Shen K, Xiong H, Wei F (2020). Imaging the node-linker coordination in the bulk and local structures of metal–organic frameworks. Nat. Commun..

[22] Ma Y (2016). The influence of straight pore blockage on the selectivity of methanol to aromatics in nanosized Zn/ZSM-5: An atomic Cs-corrected STEM analysis study. RSC Adv..

[23] Yang H, Ercius P, Nellist PD, Ophus C (2016). Enhanced phase contrast transfer using ptychography combined with a pre-specimen phase plate in a scanning transmission electron microscope. Ultramicroscopy.

[24] Pennycook TJ (2015). Efficient phase contrast imaging in STEM using a pixelated detector. Part 1: Experimental demonstration at atomic resolution. Ultramicroscopy.

[25] O'Leary CM (2020). Phase reconstruction using fast binary 4D STEM data. Appl. Phys. Lett..

[26] Lozano JG, Martinez GT, Jin L, Nellist PD, Bruce PG (2018). Low-dose aberration-free imaging of Li-rich cathode materials at various states of charge using electron ptychography. Nano Lett.

[27] Liu L (2020). Direct imaging of atomically dispersed molybdenum that enables location of aluminum in the framework of zeolite ZSM-5. Angew. Chem. Int. Ed. Engl..

[28] Hoppe W (1969). Beugung im Inhomogenen Primärstrahlwellenfeld. I. Prinzip einer Phasenmessung von Elektronenbeugungsinterferenzen. Acta Crystallogr..

[29] Rodenburg, J. & Maiden, A. in *Springer Handbook of Microscopy* (eds P. W. Hawkes & J. C. H. Spence) Ch. 17, (Springer Nature, 2019).

[30] Rodenburg JM, Bates RHT (1992). The theory of super-resolution electron microscopy via wigner-distribution deconvolution. Philos. Trans. R. Soc. Lond..

[31] Song W (2022). Direct imaging of oxygen shifts associated with the oxygen redox of Li-rich layered oxides. Joule.

[32] Maiden AM, Rodenburg JM (2009). An improved ptychographical phase retrieval algorithm for diffractive imaging. Ultramicroscopy.

[33] Humphry MJ, Kraus B, Hurst AC, Maiden AM, Rodenburg JM (2012). Ptychographic electron microscopy using high-angle dark-field scattering for sub-nanometre resolution imaging. Nat. Commun..

[34] Gerchberg RW (1972). A practical algorithm for the determination of phase from image and diffraction plane pictures. Optik.

[35] Miao J, Charalambous P, Kirz J, Sayre D (1999). Extending themethodologyof X-ray crystallography to allow imaging of micrometre-sized non-crystalline specimens. Nature.

[36] Chen Z (2021). Electron ptychography achieves atomic-resolution limits set by lattice vibrations. Science.

[37] Yang H (2016). Simultaneous atomic-resolution electron ptychography and Z-contrast imaging of light and heavy elements in complex nanostructures. Nat. Commun..

[38] Ishizuka K, Uyeda N (1977). A new theoretical and practical approach to the multislice method. Acta Crystallogr. A.

[39] Hun Kwak J, Zhu H, Lee JH, Peden CH, Szanyi J (2012). Two different cationic positions in Cu-SSZ-13?. Chem. Commun. (Camb).

[40] Borfecchia E (2015). Revisiting the nature of Cu sites in the activated Cu-SSZ-13 catalyst for SCR reaction. Chem. Sci..

[41] Rodenburg, J. M. *Advances in Imaging and Electron Physics* 87–184 (2008).

[42] Madsen J, Susi T (2021). The abTEM code: transmission electron microscopy from first principles. Open Res. Europe.

