# Supplementary material for: Direct observation of Cu in high-silica chabazite zeolite by electron ptychography using Wigner distribution deconvolution
Source: Sci Rep. 2023 Jan 6;13:316. doi: 10.1038/s41598-023-27452-3 (PMC9822938; doi:10.1038/s41598-023-27452-3)
Supplement: Supplementary file 1 — Supplementary Information. [file 41598_2023_27452_MOESM1_ESM.pdf]

Supplementary Information:

Direct observation of Cu in high-silica chabazite zeolite by electron ptychography

K. Mitsuishi[1*], K. Nakazawa[2], R. Sagawa[3], M. Shimizu[1,4], H. Matsumoto[1,4], H. Shima[5] and T. Takewaki[5]

[1]Research Center for Advanced Measurement and Characterization, National Institute for Materials Science, 1-2-1 Sengen, Tsukuba, Ibaraki 305-0047, Japan

[2]International Center for Young Scientists (ICYS), National Institute for Materials Science, 1-2-1 Sengen, Tsukuba, Ibaraki 305-0047, Japan

[3]JEOL Ltd., 3-1-2 Musashino, Akishima, Tokyo 196-8558, Japan

[4]Materials Characterization Laboratory, Mitsubishi Chemical Corporation, 1000 Kamoshida-cho, Aoba-ku, Yokohama-shi, Kanagawa, Japan

[5]Science & Innovation Center, Mitsubishi Chemical Corporation, 1000 Kamoshida-cho, Aoba-ku, Yokohama-shi, Kanagawa, Japan

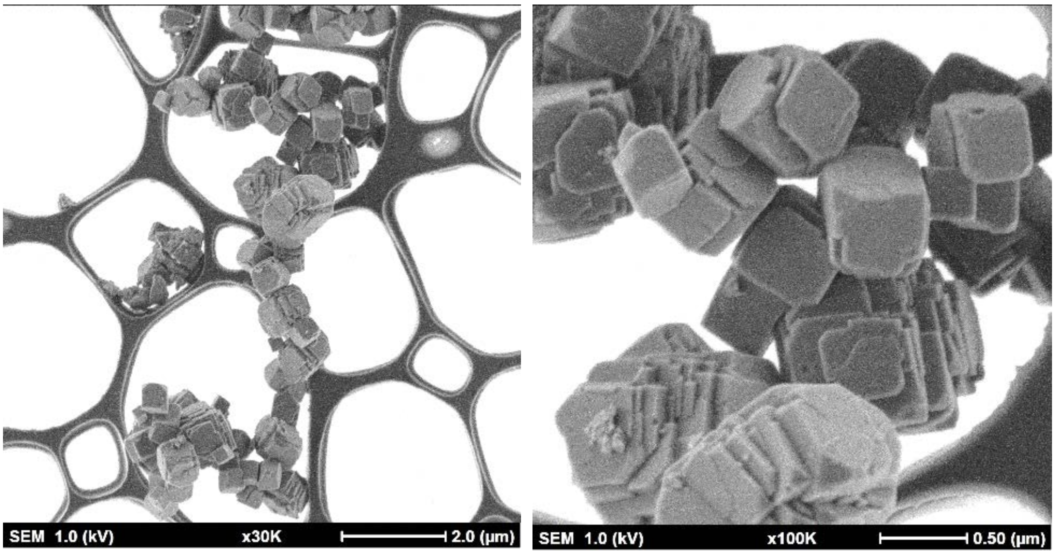

Figure S1 SEM images of Cu-SSZ-13 used for TEM observations.

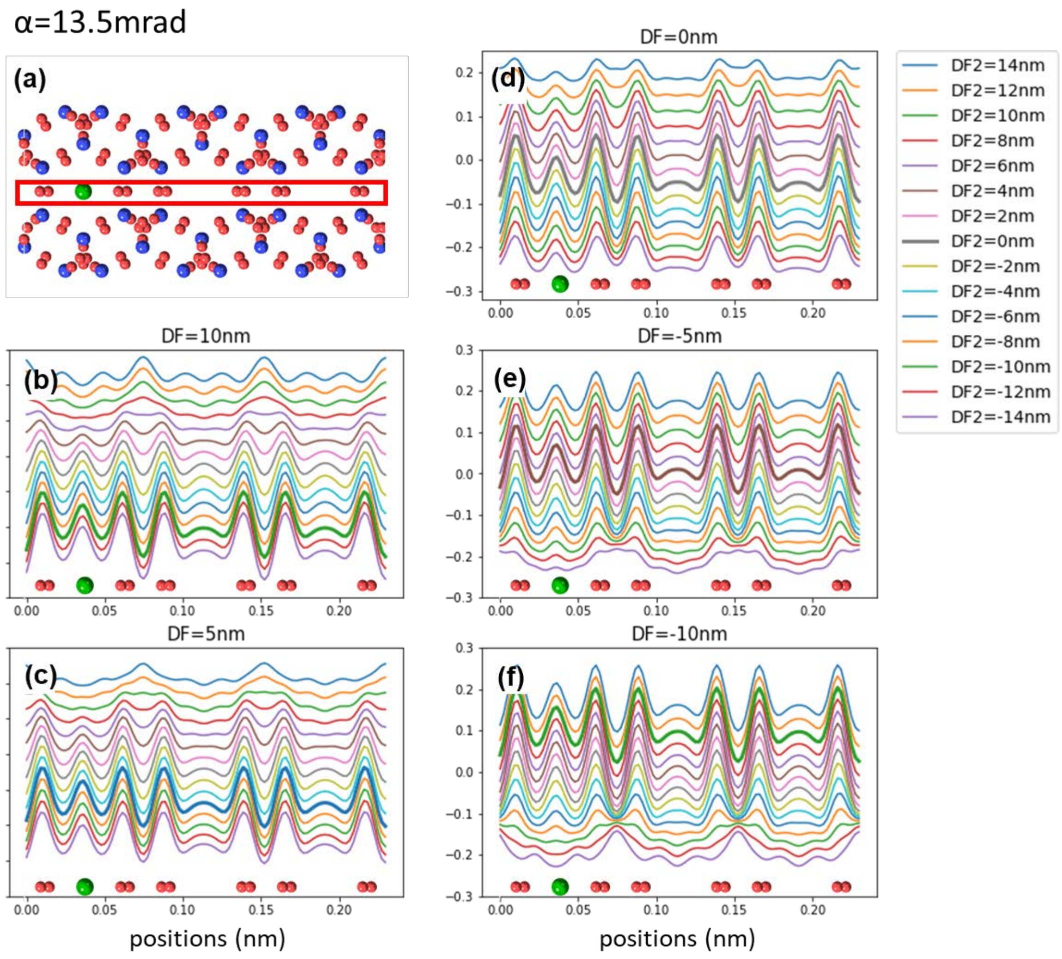

Figure S2 Line profiles of the simulated images along the columns with and without Cu for a convergence semi-angle of 13.5 mrad. (a) Model structure, in which the red rectangle indicates the position of the line profile. (b)–(f) Line profiles for acquisition defocus values of (b) 10 nm, (c) 5 nm, (d) 0 nm, (e) −5 nm, and (f) −10 nm and reconstruction defocus values from −14 to 14 nm (shown as DF2 in the legend). The thick lines correspond to the reconstruction defocus (DF2) that compensates for the acquisition defocus (DF). The lines are vertically shifted for display purposes

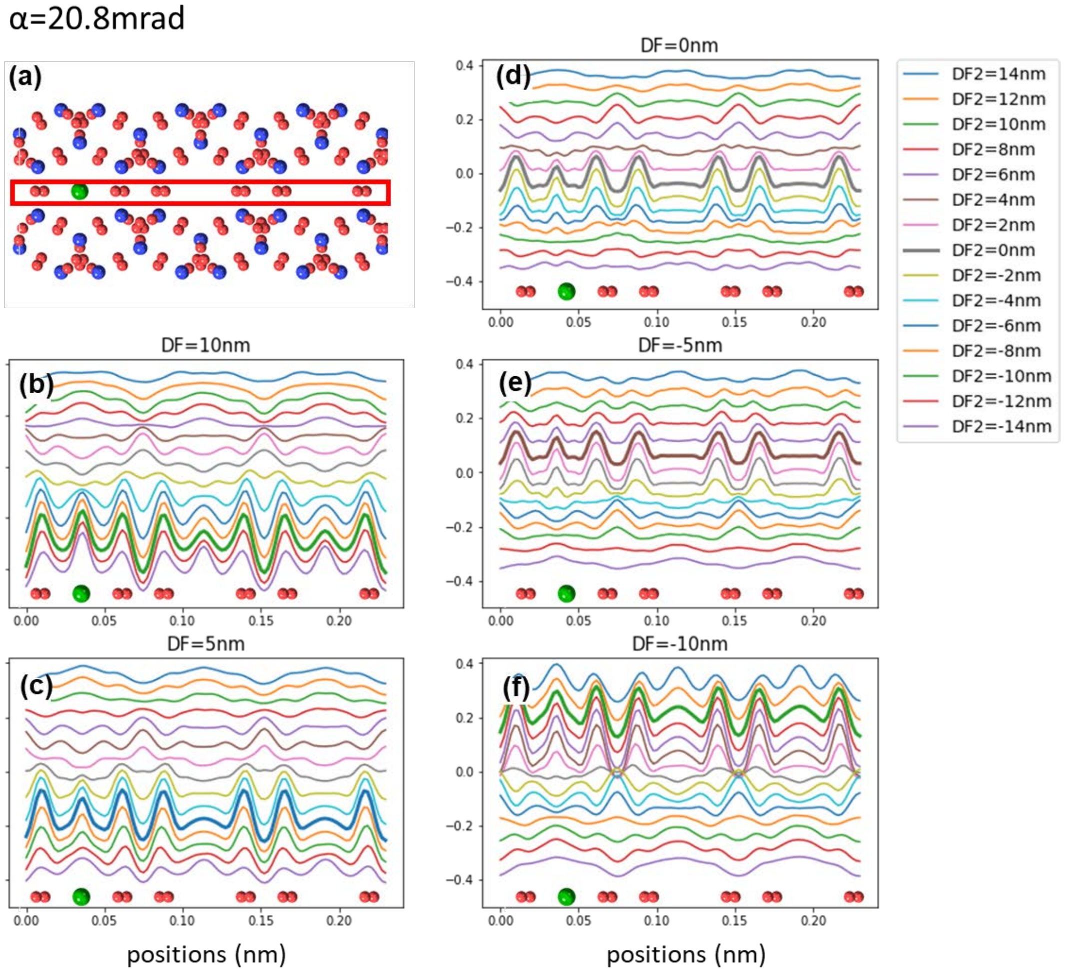

Figure S3 Line profiles of the simulated images along the columns with and without Cu for a convergence semi-angle of 20.8 mrad. (a) Model structure, in which the red rectangle indicates the position of the line profile. (b)–(f) Line profiles for acquisition defocus values of (b) 10 nm, (c) 5 nm, (d) 0 nm, (e) −5 nm, and (f) −10 nm and reconstruction defocus values from −14 to 14 nm (shown as DF2 in the legend). The thick lines correspond to the reconstruction defocus (DF2) that compensates for the acquisition defocus (DF).

The lines are vertically shifted for display purposes

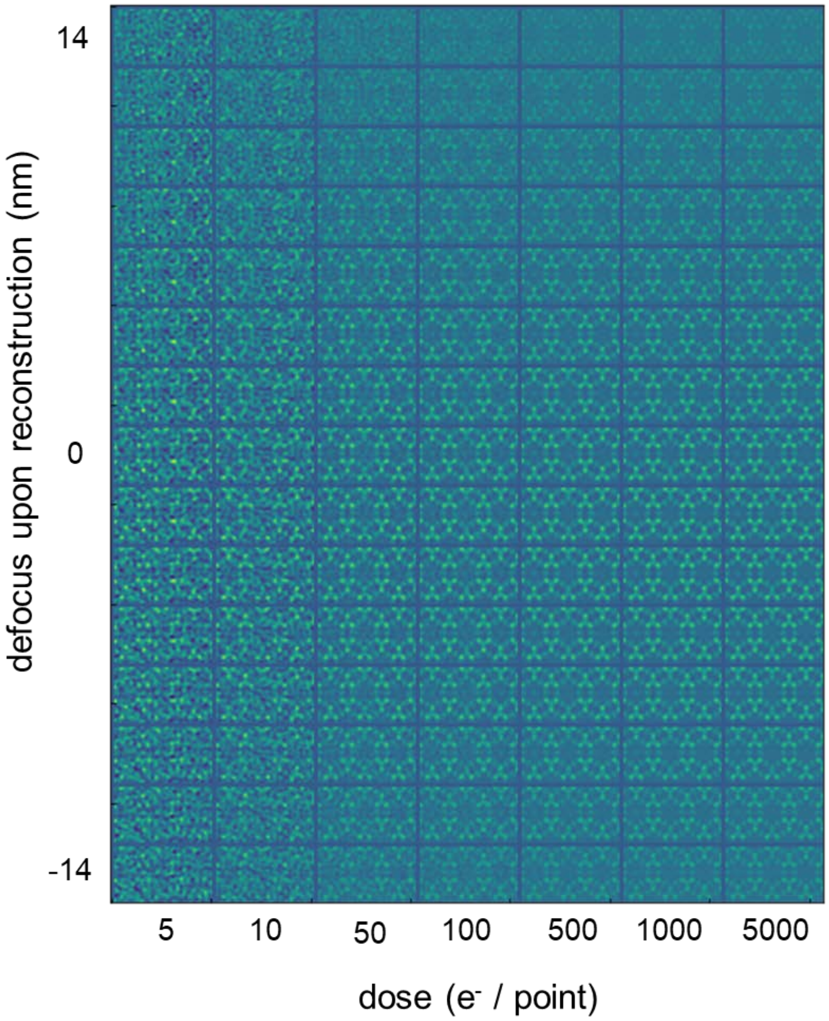

Figure S4. Through-focus image simulation for reconstruction defocus with different electron doses ranging from 5 to 5000 electrons per scan point. The simulated region of 2.3 × 1.3 nm corresponds to the black rectangular region in Fig. 1(a) containing six-membered-ring columns with (left) and without (center and right) Cu. The convergence semi-angle was 13.5 mrad and the acquisition defocus was fixed at zero. The relation between e^{-}/point and $e^{-}/Å^{2}$ is as follows: a dose of 100 e^{-}/point with 512 × 512 scan points for 10 × 10 nm scan area results in 2600 $e^{-}/Å^{2}$.

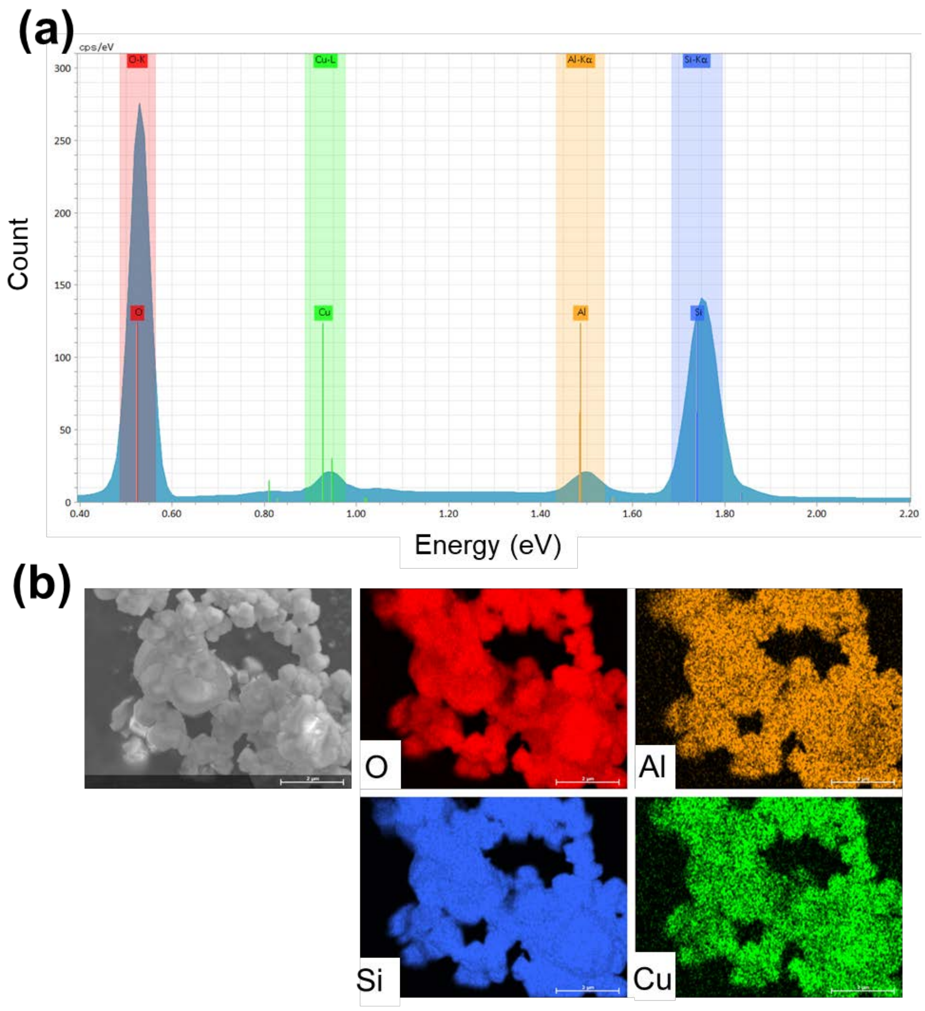

Figure S5. SEM-EDS analysis for a Cu ion-exchanged sample. (a) EDS spectra and (b) SEM image and elemental maps. We used a Hitachi SU5000 SEM equipped with a QUANTAX FlatQUAD EDS detector from Bruker at an acceleration voltage of 4 kV.

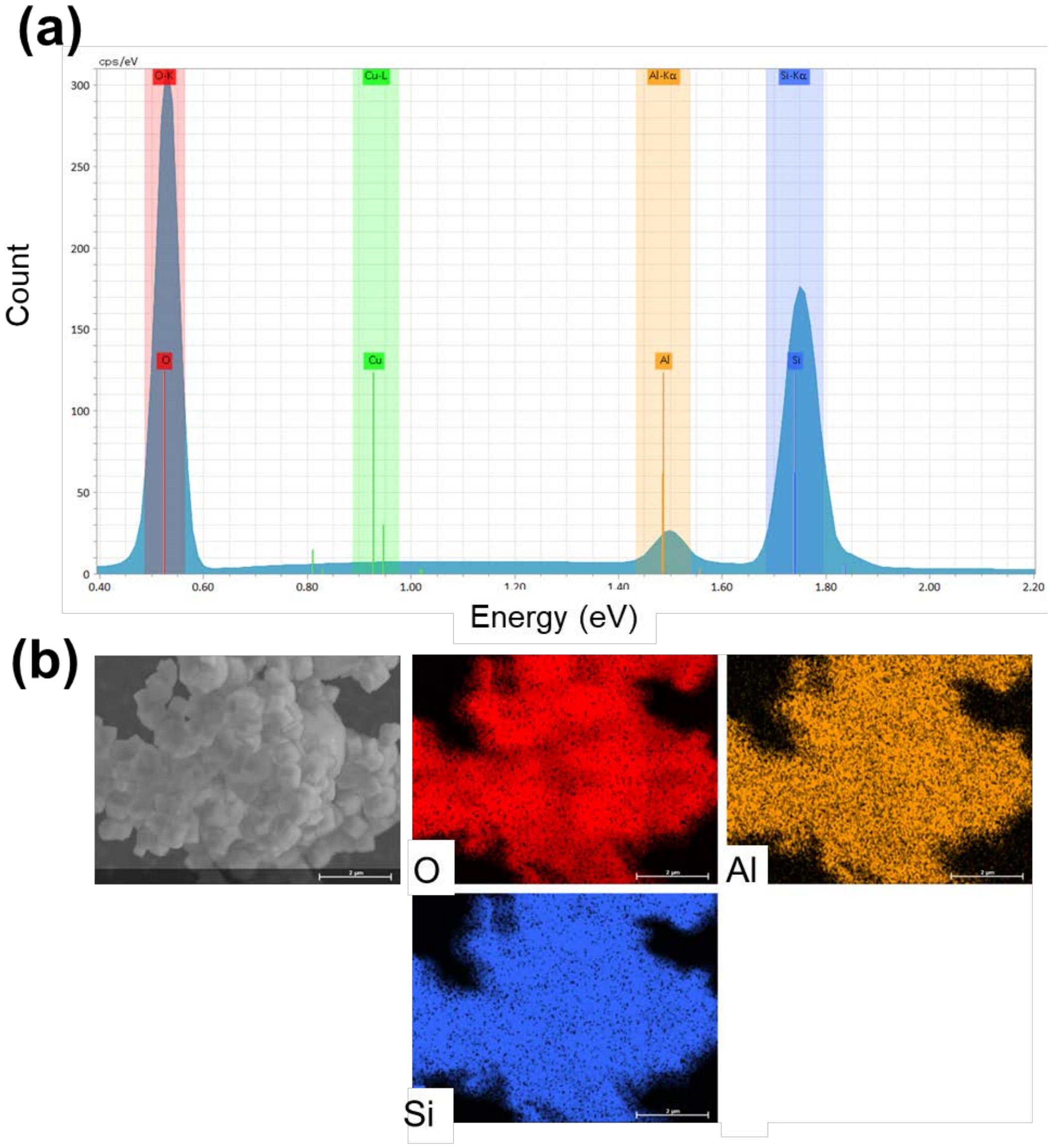

Figure S6. SEM-EDS analysis for a proton-exchanged sample. (a) EDS spectra and (b)SEM image and elemental maps.

Table S1. Measured values of the aberration coefficients

Aberration Coefficients		Measured values for Figs. 5 and 6.	Measured values for Figs. 7 and 8.
Defocus	C_1	-1.68 (nm)	+1.50 (nm)
Two-fold astigamatism	C_{12a}	+1.21 (nm)	-2.86 (nm)
Two-fold astigamatism	C_{12b}	+0.80 (nm)	-4.10 (nm)
Three-fold astigamatism	C_{23a}	+1.36 (nm)	-9.56 (nm)
Three-fold astigamatism	C_{23b}	-24.4 (nm)	-8.33 (nm)
Axial coma	C_{21a}	-145 (nm)	-217 (nm)
Axial coma	C_{21b}	-21.5 (nm)	-123 (nm)
Spherical aberration	C_3	+7.10 (μm)	1.72 (μm)
Four-fold astigmatism	C_{34a}	-3.50 (μm)	+0.53 (μm)
Four-fold astigmatism	C_{34b}	-4.53 (μm)	-8.44 (μm)
Star aberration	C_{32a}	+3.47 (μm)	-3.06 (μm)
Star aberration	C_{32b}	-5.00 (μm)	+0.34 1 (μm)
